# Supplementary material for: MetaRibo-Seq measures translation in microbiomes
Source: Nat Commun. 2020 Jun 29;11:3268. doi: 10.1038/s41467-020-17081-z (PMC7324362; doi:10.1038/s41467-020-17081-z)
Supplement: Supplementary file 10 — Supplementary Data 7 [file 41467_2020_17081_MOESM10_ESM.zip › File2/Confidence_High_Taxonomy/165703_out.krona.html]

Javascript must be enabled to view this page.

members
magnitude
magnitudeUnassigned
count
unassigned
taxon
rank

165703\_out

11

2
superkingdom
11

11
phylum
976

11
class
200643

171549
order
11

11
family
2005525

375288
genus
11


SRS043768\_contig\_number\_813
species
1
387661


SRS048870\_contig\_number\_13530SRS049959\_contig\_number\_51696SRS050422\_contig\_number\_contig-100\_894.175993SRS050520\_contig\_number\_contig-100\_373.374SRS065397\_contig\_number\_5738SRS075773\_contig\_number\_35915SRS1041130\_contig\_number\_4405SRS146813\_contig\_number\_contig-100\_811.163280SRS893170\_contig\_number\_contig-100\_1173.63211SRS893230\_contig\_number\_contig-100\_1174.72521
species
10
46503
